# The potential epidemiologic, clinical, and economic value of a universal coronavirus vaccine: a modelling study

**DOI:** 10.1016/j.eclinm.2023.102369

**Published:** 2024-01-11

**Authors:** Sarah M. Bartsch, Kelly J. O'Shea, Danielle C. John, Ulrich Strych, Maria Elena Bottazzi, Marie F. Martinez, Allan Ciciriello, Kevin L. Chin, Colleen Weatherwax, Kavya Velmurugan, Jessie Heneghan, Sheryl A. Scannell, Peter J. Hotez, Bruce Y. Lee

**Affiliations:** a Public Health Informatics, Computational, and Operations Research (PHICOR), CUNY Graduate School of Public Health and Health Policy, New York City, NY, USA; b Center for Advanced Technology and Communication in Health (CATCH), CUNY Graduate School of Public Health and Health Policy, New York City, NY, USA; c Pandemic Response Institute, New York City, NY, USA; d National School of Tropical Medicine, Department of Pediatrics, and Texas Children's Hospital Center for Vaccine Development, Baylor College of Medicine, Houston, TX, USA; e Department of Molecular Virology and Microbiology, Baylor College of Medicine, Houston, TX, USA

**Keywords:** Coronavirus, Vaccine, Universal, Economic, Modelling

## Abstract

**Background:**

With efforts underway to develop a universal coronavirus vaccine, otherwise known as a pan-coronavirus vaccine, this is the time to offer potential funders, researchers, and manufacturers guidance on the potential value of such a vaccine and how this value may change with differing vaccine and vaccination characteristics.

**Methods:**

Using a computational model representing the United States (U.S.) population, the spread of SARS-CoV-2 and the various clinical and economic outcomes of COVID-19 such as hospitalisations, deaths, quality-adjusted life years (QALYs) lost, productivity losses, direct medical costs, and total societal costs, we explored the impact of a universal vaccine under different circumstances. We developed and populated this model using data reported by the CDC as well as observational studies conducted during the COVID-19 pandemic.

**Findings:**

A pan-coronavirus vaccine would be cost saving in the U.S. as a standalone intervention as long as its vaccine efficacy is ≥10% and vaccination coverage is ≥10%. Every 1% increase in efficacy between 10% and 50% could avert an additional 395,000 infections and save $1.0 billion in total societal costs ($45.3 million in productivity losses, $1.1 billion in direct medical costs). It would remain cost saving even when a strain-specific coronavirus vaccine would be subsequently available, as long as it takes at least 2–3 months to develop, test, and bring that more specific vaccine to the market.

**Interpretation:**

Our results provide support for the development and stockpiling of a pan-coronavirus vaccine and help delineate the vaccine characteristics to aim for in development of such a vaccine.

**Funding:**

The 10.13039/100000001National Science Foundation, the 10.13039/100000133Agency for Healthcare Research and Quality, the 10.13039/100000057National Institute of General Medical Sciences, the 10.13039/100006108National Center for Advancing Translational Sciences, and the 10.13039/100006462City University of New York.

**Research in context**

**Evidence before this study**

With efforts underway to develop a universal coronavirus vaccine, otherwise known as a pan-coronavirus vaccine, this is the time to offer potential funders, researchers, and manufacturers guidance on the potential value of such a vaccine and how this value may change with differing vaccine and vaccination characteristics. To review the existing literature, we searched MEDLINE via PubMed for studies published through March 2023 using search terms such as “pan-coronavirus vaccine,” “universal coronavirus vaccine,” “sarbecovirus vaccine,” “betacoronavirus vaccine.” Previous studies have focused on the technical aspects of vaccine development (e.g., biological targets for pan-coronavirus immunity). To our knowledge, studies have not yet evaluated the epidemiologic, clinical, and economic value of such a universal coronavirus vaccine.

**Added value of this study**

In order to determine the potential impact and value of a universal coronavirus vaccine in the event of a new coronavirus epidemic, we utilised a computational simulation model of the U.S. representing the spread of a new coronavirus and its subsequent clinical and economic outcomes. We found that a pan-coronavirus vaccine would be cost saving as a standalone intervention as long as the vaccine efficacy is ≥10% and vaccination coverage is ≥10%. It would remain cost saving even when a strain-specific coronavirus vaccine would be subsequently available, as long as this specific vaccine is delayed by at least 2–3 months.

**Implications of all the available evidence**

Our results can help a variety of decision makers like funders, researchers, and vaccine manufacturers as our study provides support for the development and stockpiling of a universal (pan-coronavirus) vaccine and helps delineates the vaccine characteristics to aim for in development of such a vaccine, which can provide targets for vaccine manufactures.

## Introduction

While efforts are underway to develop a universal coronavirus vaccine, otherwise known as a pan-coronavirus vaccine, potential funders, researchers, and manufacturers could use guidance on the potential value of such a vaccine and how this value may change with differing vaccine and vaccination characteristics.^1^^,^^2^ Severe acute respiratory virus 2 (SARS-CoV-2) is the third major human coronavirus infection to emerge in the 21st century, after severe acute respiratory syndrome (SARS) in 2002 and Middle East Respiratory Syndrome (MERS) in 2012.^3^^,^^4^ This suggests that another novel coronavirus is likely to emerge in the near future, potentially leading to another outbreak, epidemic, or even pandemic. Rather than simply wait for another such coronavirus to emerge, it may be beneficial to develop and stockpile a pan-coronavirus vaccine that could provide at least partial protection against all viruses in the genus betacoronavirus, including the subgenera sarbecoviruses (e.g., SARS-CoV and SARS-CoV-2) and merbecoviruses (e.g., MERS-CoV).

If universal coronavirus vaccines had existed prior to the start of the COVID-19 pandemic in 2020, such vaccines could have potentially saved lives and prevented suffering during the 10 months before vaccines that were more specific to that type of coronavirus (i.e., SARS-CoV-2) managed to go through the entire development, testing, and authorisation process that lasted until December 2020^5^. In fact, given the high mutation rate seen among many coronaviruses,^6^^,^^7^ a universal vaccine may even be preferable to more specific coronavirus vaccines that may have to be updated regularly when significantly different variants and sub-variants emerge.^8^^,^^9^ Quantifying the potential value of such universal vaccines with varying characteristics under different circumstances can help guide how much should be invested into developing and stockpiling them, what characteristics should be aimed for in developing such universal vaccines (e.g., developing target product profiles), and how such universal vaccines should be deployed.^10^, ^11^, ^12^, ^13^, ^14^ Therefore, we utilised a computational simulation model of the U.S. to represent the spread and impact of different coronaviruses and evaluate the various types of universal and strain-specific coronavirus vaccines.

## Methods

### Model of the U.S. population

**Fig. 1:**
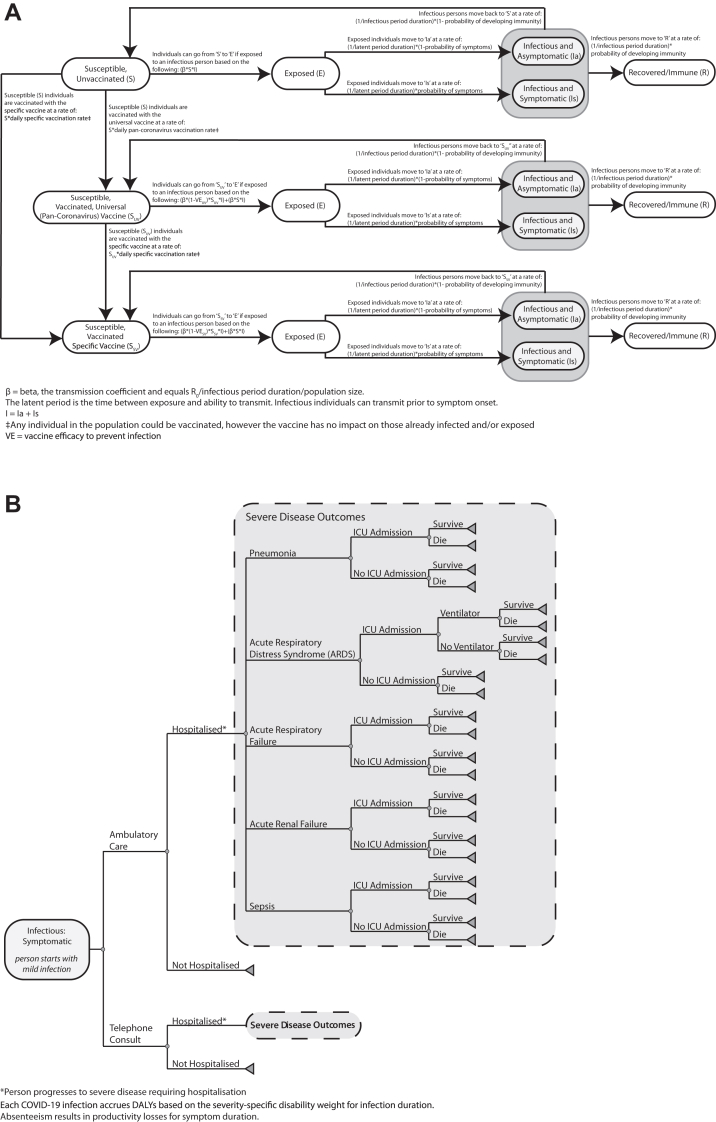
Model Structure A) SARS transmission; and B) clinical outcomes probability tree.

We adapted our previously described SARS-CoV-2 computational model^12^, ^13^, ^14^, ^15^, ^16^, ^17^, ^18^ (developed in Microsoft Excel with the Crystal Ball add-in) to represent the spread of a new virus strain from the SARBECO (SARS beta coronavirus) family in the U.S. The population is divided into three age groups: children (≤17 years old), adults (18–64 years old), and older adults (≥65 years old), following the 2021 U.S. Census.^19^ Each group has different mixing patterns with each other following previous studies.^20^^,^^21^ Appendix Table S1 shows the key model input parameters, values, and sources. Fig. 1A shows the model structure, how people mix with each other, the different mutually exclusive compartments that each person can be in on a given simulated day, and the equations that govern how and when individuals move among them. For example, an individual can move from being susceptible (S) to exposed (E) when he/she interacts with an infectious individual (either I_a_ or I_s_, based on symptoms) based on the following equation: β∗S∗(I_s_ + I_a_) where β is the transmission coefficient and equals the reproduction number of the virus (R_0_; average number of secondary cases generated by one infectious case in a completely susceptible population) divided by the infectious period duration divided by the population size. A higher R_0_ suggests the virus has greater transmission, which may mean the infected individual may spread more of the virus, or the virus has greater durability/can survive longer in the air, or it may be more likely to enter cells causing infection.

The Appendix includes a description of the model validation process. To be conservative about the value of vaccination, we assume that protection from natural immunity does not wane over time.

### Clinical and economic submodel within each person

Each time a person in the model gets infected, he/she proceeds through a clinical and economic outcomes submodel (Fig. 1B) that has been described in previous publications.^12^, ^13^, ^14^, ^15^, ^16^, ^17^, ^18^ As each individual develops different clinical manifestations of the infection, this individual accrues the corresponding health effects, productivity losses, and direct medical costs. These then contributed to the calculation of cost-benefit and cost-effectiveness of pan-coronavirus vaccination from different perspectives such as that of the third-party payer (direct medical costs such as vaccination and hospitalisation) and societal (direct medical costs plus productivity losses) perspectives. The following formula calculated health effects in disability-adjusted life-years (DALYs):

DALYs = YLLs + YLDs = Year of Life Lost Due to Premature Mortality + Years of Life Lived With Disability = (Number of Cases x Disability Weight x Duration of Disability in Years) + (Number of Deaths x Life Expectancy at Age of Death in Years).

### Pan-coronavirus vaccine

We represent a pan-coronavirus vaccine that is already stockpiled and available at the beginning of the epidemic and provides partial protection against the circulating coronaviruses. As previously described,^12^, ^13^, ^14^, ^15^, ^16^, ^17^, ^18^ receiving a vaccine decreases the risk of an individual getting infected by 1 minus the pan-coronavirus vaccine efficacy to prevent infection. Once infected, a vaccinated individual has a lower probability of developing severe outcomes (1-vaccine efficacy to prevent severe disease). Protection onset (i.e., the time point at which protection begins) occurs 2 weeks post-vaccination, after which individuals have the full starting efficacy of the pan-coronavirus vaccine. We assumed the vaccine has the same efficacy against infection and severe disease. However, we assume that only vaccine efficacy against infection can wane (i.e., decrease) over time. When it wanes, the pan-coronavirus vaccine efficacy remains stable until month 4 when it starts to decrease in a straight line (e.g., linearly) to its ending efficacy at month 6, which is held for the remainder of the simulation (similar to current COVID-19 vaccines^22^^,^^23^).

### Coronavirus strain-specific vaccine

We also represent a more specific coronavirus vaccine that is not immediately available, will take time to develop, test, and reach the market, but is more specific to the circulating coronavirus as it is made after the stain has been identified and thus likely to have a higher efficacy. Again, the strain-specific vaccine decreases the risk of an individual getting infected by 1 minus the strain-specific vaccine efficacy once infected, a vaccinated individual has a lower probability of developing severe outcomes (1-strain-specific vaccine efficacy). Protection onset for the strain-specific vaccine occurs 2 weeks post-vaccination and efficacy does not wane over time.

### Experimental scenarios

We ran the following experimental scenarios:

• *No vaccines available*: these scenarios assume that no vaccines are available throughout the epidemic and that no other interventions (e.g., social distancing, face mask use) are implemented.
• *The pan-coronavirus vaccine is the only available vaccine*: in these scenarios, a pan-coronavirus vaccine is stockpiled and available to be administered at the start of the new epidemic. The entire population is eligible for vaccination (as there are few restrictions for the currently available COVID-19 vaccines^24^). Individuals can receive the pan-coronavirus vaccine starting on day one of the simulation and are vaccinated each day, based on the daily vaccination rate, until achieving the total vaccination coverage level. This vaccine has an associated efficacy and can either wane or not wane over time (described above).
• *The pan-coronavirus vaccine is available at the beginning of the epidemic and a more strain-specific coronavirus vaccine becomes available later*: these scenarios introduce a strain-specific vaccine that is delayed for different times from the epidemic start (to account for different timings for vaccine development, testing, and deployment once the strain is identified). Once available, individuals could receive this strain-specific vaccine, regardless of whether they received the pan-coronavirus vaccine or not. Individuals are vaccinated each day, based on the daily vaccination rate, until reaching the total strain-specific vaccination coverage level. Again, the entire population is eligible for vaccination and individuals are vaccinated with the strain-specific vaccine each day until its target coverage is reached. To be conservative about the value of a pan-coronavirus vaccine, we assume that the strain specific vaccine efficacy does not wane and that everyone who is infected becomes immune (i.e., 100% seroconvert).

The initial base case scenario assumed that the new coronavirus epidemic would be similar to that of the one caused by the original/Wuhan strain in 2020 in terms of transmission (R_0_ 2.5) and clinical severity. Additional scenarios simulated other types of possible coronavirus epidemics by varying:

• *R*_*0*_*of the virus:* from 2.5 to 9 to account for different possible variants/mutations
• *Probability of different clinical outcomes:* ranging the probability of severe clinical outcomes seen in 2020 to 2 times these probabilities

Sensitivity analyses explored the impact of varying:

• *Efficacy of the pan-coronavirus vaccine:* ranging from 10% to 70% to account for different possible starting efficacies as well as possible virus variants/mutations
• *Waning of the pan-coronavirus vaccine efficacy:* from no waning efficacy to waning to 0% over 6 months (described above)
• *Vaccination coverage of the pan-coronavirus vaccine:* ranged from 10% to 50% to account for different vaccination strategies as well as different levels of acceptance and willingness to get vaccinated (e.g., hesitancy)
• *How long it takes to achieve coverage with the pan-coronavirus vaccine:* 1–3 months
• *Efficacy of the strain-specific vaccine:* 50%–90% to account for different possible strains
• *How long the strain-specific vaccine is delayed:* varied from 2 to 6 months
• *Vaccination coverage of the strain-specificvaccine:* varied from 50% to 70% to account for different vaccination strategies, levels of acceptance, and willingness to get vaccinated

Each simulation experiment consists of running the model 1000 times (i.e., Monte Carlo simulations), varying each parameter across their distribution (Appendix Table S1). In a given simulation, the model progresses until there are fewer than 100 cases. For each scenario, the following formula calculated the incremental cost-effectiveness ratio (ICER) of the universal vaccine:

ICER = (Cost_Pan-CoronavirusVaccine_-Cost_NoPan-CoronavirusVaccine_)/(Health Effects_NoPan-CoronavirusVaccine_-HealthEffects_Pan-CoronavirusVaccine_)

Use of the pan-coronavirus vaccine is considered highly cost-effective when the ICER is less than the gross domestic product (GDP) per capita, which is $78,691 for the U.S. in 2023 values,^25^ cost-effective when 1–3 times the GDP, and cost saving when less than $0. We used a 3% annual rate to convert all costs to 2023 values.^26^ A paired t-test performed in Microsoft Excel (Redmond, WA) determined the statistical significance between the pan-coronavirus vaccine and either no vaccines available or strain specific-vaccines become available scenarios. Our study adhered to the CHEERS Value of Information (CHEERS-VOI) Reporting Standards.^27^

### Role of the funding source

The funder of the study had no role in study design, data collection, data analysis, data interpretation, or writing of the report. Authors SMB, KJO, DCJ, KLC, CW, KV, JH, and BYL had access to the dataset. BYL had final responsibility for the decision to submit for publication.

## Results

### How a Pan-coronavirus vaccine could have helped in 2020

**Fig. 2:**
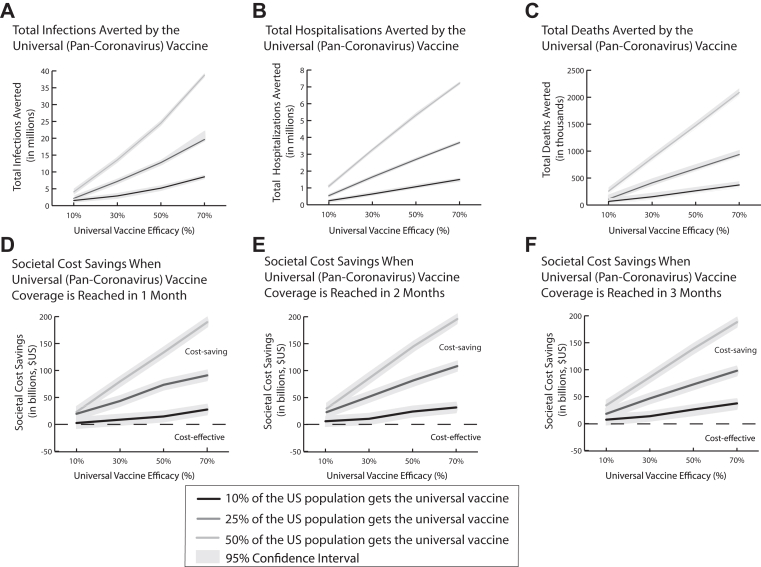
Clinical outcomes (mean, 95% confidence interval) averted (A–C) and societal cost savings yielded (D–F) by using a universal coronavirus vaccine (costing $60) when it is the only intervention available and how it varies with vaccine efficacy (assuming it wanes to 0% at 6 months) and coverage.

Fig. 2 shows how many coronavirus infections, hospitalisations, and deaths could have been averted in addition to how much societal costs could have been saved had a pan-coronavirus vaccine been available as a standalone intervention in a pandemic similar to 2020 conditions (R_0_ 2.5) and how these amounts increase with vaccine efficacy and coverage. As shown in Fig. 2, when 25% of the U.S. population is vaccinated within 2 months, for every 1% increase in efficacy between 10% and 50% (where efficacy does not wane), the pan-coronavirus vaccine averts an additional 395,000 infections, 54,000 hospitalisations, and 12,600 deaths, and saves an additional $1.1 billion in direct medical costs, $45.3 million in productivity losses, and $1.0 billion in societal costs (Fig. 2). A pan-coronavirus vaccine with efficacy as low as 10% that does not wane is cost saving, saving $27.8 billion (95% confidence interval [CI]: $26.8–28.9 billion) in direct medical costs, $18.3 billion (95% CI: $8.4–28.3 billion) in productivity losses, and $41.2 billion (95% CI: $31.2–51.2 billion) in societal costs, and averting on average 14.62 million (95% CI: 14.60–14.65 million) infections, 1.28 million (95% CI: 1.23–1.34 million) hospitalisations, 403,000 (95% CI: 353,000–453,000) deaths, and 23.0 million (95% CI: 19.4–26.6 million) DALYs, all of which are statistically significant (*p*-values ≤0.0003). Even when its efficacy wanes to 0% over 6 months, the pan-coronavirus vaccine remains cost saving with a starting efficacy as low as 10%.

Furthermore, even when coverage only reaches 10% over 2 months (Fig. 2E), a pan-coronavirus vaccine with a ≥10% efficacy still saves at least $4.2 billion (95% CI: $3.2–$5.3 billion) in direct medical costs, $10.2 billion (95% CI: $0.27–$20.1 billion) in productivity losses, and $12.4 billion (95% CI: $2.4–22.4 billion) in societal costs (*p*-values ≤0.044). This could be realised when vaccination is only indicated for certain higher-risk subpopulations such as those who are older and/or have other reasons to have weaker immune systems such as pre-existing health conditions.

**Fig. 3:**
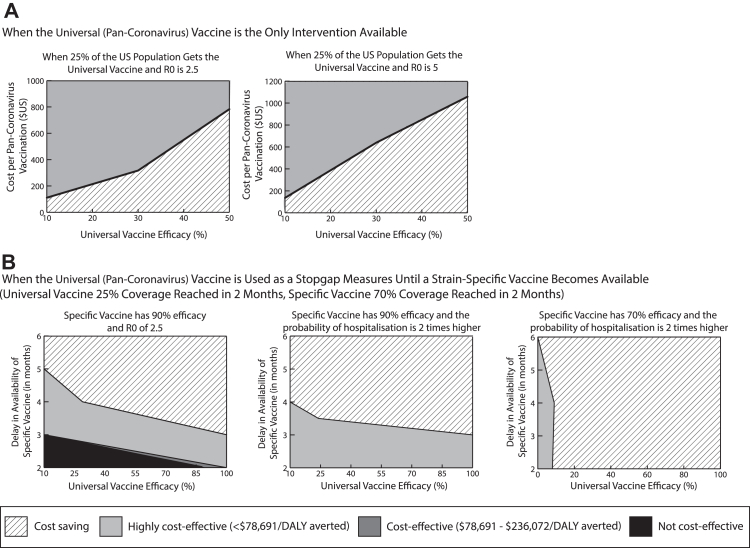
Vaccination cost and vaccine efficacy thresholds at which a universal coronavirus vaccine is cost saving and cost-effective (cost per disability-adjusted life year averted) from the societal perspective when A) the universal vaccine is the only intervention available; and B) when it is used as a stopgap measure prior to a strain-specific vaccine becoming available.

Fig. 3A is a threshold map that shows at which combinations of pan-coronavirus vaccine efficacy and vaccination cost using a pan-coronavirus vaccine becomes highly cost-effective (<$78,691/DALY averted) and cost saving. For example, when 25% of the population gets a vaccine with an efficacy of 10%, it is cost saving up to a vaccination cost of $110 and highly cost-effective up to $10,390. These cost thresholds increase by 1.4–2.8 times with higher efficacies (e.g., $315 and $14,500 with a 30% vaccine efficacy).

### How a pan-coronavirus coronavirus vaccine can help in future outbreaks as a standalone intervention

In order to determine the potential impact of a pan-coronavirus vaccine in future outbreaks, these experiments range different viral characteristics such as the transmissibility (R_0_) of the virus and the probability of more severe clinical outcomes (e.g., hospitalisation, death). Increasing R_0_ from 2.5 to 5 (similar to June–November 2021 when the delta variant was predominant) decreases the number of infections averted because the virus spreads more rapidly through the population, but increases the number of hospitalisations, deaths, and DALYs averted since the vaccine can potentially avert more of these outcomes as there are more cases overall; in general, this increases the resulting cost savings of universal vaccination as well as the cost thresholds. For example, even when the pan-coronavirus vaccine has a 30% efficacy that wanes to 0%, vaccinating 25% of the U.S. population within 2 months averts on average 6 million (95% CI: 5.9–6.0 million) infections, 2.2 million (95% CI: 2.1–2.2 million) hospitalisations, 592,300 (95% CI: 509,000–675,400) deaths, and 39.6 million (95% CI: 33.5–45.8 million) DALYs, saving $46.3 billion (95% CI: $45.0–47.6 billion) in direct medical costs, $18.7 billion (95% CI: $6.3–31.0 billion) in productivity losses, and $64.9 billion (95% CI: $52.5–77.3 billion) in societal costs (all *p-*values ≤0.0031). Additionally, the pan-coronavirus vaccination cost thresholds increase and are 1.2–2.1 times higher than those when R_0_ is 2.5 (e.g., cost saving up to $640 and highly cost-effective up to $29,900 with a 30% vaccine efficacy; Fig. 3A).

Similarly, higher risks of severe outcomes only increase the value of a pan-coronavirus vaccine. For example, when people are 1.5 times more likely to be hospitalised, even using a pan-coronavirus vaccine with an efficacy as low as 10% that wanes to 0% averts an average of 7.0 million (95% CI: 6.9–7.1 million) hospitalisations, 2.2 million (95% CI: 2.1–2.3 million) deaths, and 148.6 million (95% CI: 140.9–156.2 million) DALYs, and saves $146.0 billion in (95% CI: $144.1–148.0 billion) direct medical costs, $16.8 billion (95% CI: $2.7–26.9 billion) in productivity losses, and $133.9 billion (95% CI: $123.4–144.4 billion) in societal costs (*p*-values ≤0.0011).

### How a pan-coronavirus vaccine can help when a strain-specific coronavirus vaccine will be available after a delay assuming 2020 conditions

**Fig. 4:**
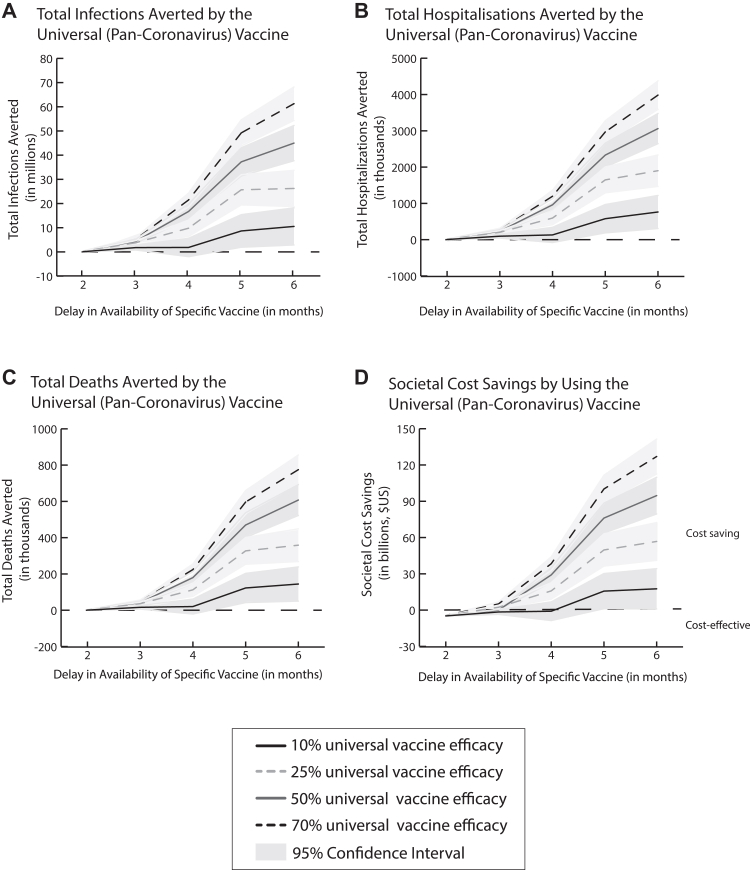
Clinical outcomes (mean, 95% confidence interval) averted (A–C) and societal cost savings yielded (D) by using a universal coronavirus vaccine (25% coverage in 2 months) when a strain-specific vaccine (90% efficacy, 70% coverage in 2 months) does not become available until later in the pandemic.

When a specific vaccine targeted towards the circulating strain becomes available later in a pandemic similar to 2020 conditions (similar to what was seen with the COVID-19 pandemic when specific vaccines came out at the end of 2020), using a pan-coronavirus vaccine with an efficacy as low as 10% that wanes as a stopgap measure (i.e., a temporary intervention prior to deploying a better one) is highly cost-effective and even cost saving, depending on the specific vaccine delay and efficacy. For example, when a specific vaccine with an efficacy of 90% is delayed 3 months after the pandemic start (70% coverage achieved in 2 months), vaccinating 25% of the population with a 10% efficacious pan-coronavirus vaccine that wanes at the beginning of the pandemic is highly cost-effective ($44,174/DALY averted from the societal perspective). Such a pan-coronavirus vaccine becomes cost saving if the specific vaccine is delayed ≥4 months (Fig. 3B). Fig. 4 further shows how the number of clinical outcomes averted and societal cost savings yielded by a pan-coronavirus vaccine increase more than linearly as the delay in the availability of the specific vaccine increases and the epidemic curve starts to rise sharply, which increases the number of cases that a universal vaccine can potentially avert.

Decreasing the specific vaccine efficacy only makes the pan-coronavirus vaccine more clinically and economically valuable. For example, when the specific vaccine is only 50% efficacious, a pan-coronavirus vaccine with 10% efficacy that wanes is highly cost-effective ($2139/DALY averted) when the specific vaccine is delayed by at least 2 months and becomes cost saving with specific vaccine delays ≥2.5 months. Similarly, further delaying the availability of the specific vaccine only increases the value of the pan-coronavirus vaccine. For example, if this specific vaccine is delayed by 6 months, vaccinating 25% of the population with a pan-coronavirus vaccine (10% efficacy that wanes) saves $16.2 billion (95% CI: $6.1–26.4 billion) in societal costs compared to waiting for the specific vaccine (*p* = 0.0018).

When the specific vaccine's vaccination coverage is closer to that observed in the COVID-19 pandemic (e.g., 50%), a pan-coronavirus vaccine is cost saving even at coverages as low as 10%. For example, if the pan-coronavirus vaccine achieves a 10% coverage in 2 months (10% efficacy) and a 90% efficacious specific vaccine becomes available at 2 months and achieves 50% coverage, the pan-coronavirus vaccine saves $2.6 billion in total societal costs.

### How a pan-coronavirus vaccine can help when a more specific coronavirus vaccine will be available after a delay under other potential conditions

When the transmissibility of the virus or the severity of the resulting clinical outcomes increases, the pan-coronavirus vaccine becomes cost saving at very low efficacies with shorter specific vaccine delays. For example, when R_0_ goes from 2.5 to 5, the specific vaccine delay threshold at which a 10% efficacious pan-coronavirus vaccine becomes cost saving drops from 4 months to only 2 months (specific vaccine efficacy 90%, 70% vaccination coverage). At this threshold (25% coverage), the pan-coronavirus vaccine averts on average 4.7 million (95% CI: −0.98 to 9.5 million) infections, 456,000 (95% CI: 140,700–771,000) hospitalisations, and 85,000 (95% CI: 10,020–160,300) deaths, and 5.7 million (95% CI: 0.6–11 million) DALYs, saving $9.6 billion (95% CI: $2.8–16.4 billion) in direct medical costs and $8.1 billion (95% CI: −$4.0 to 20.1 billion) in societal costs (*p*-values: 0.0046–0.19).

Similarly, doubling the risk of hospitalisation from what was seen in 2020 for COVID-19 decreases the efficacy thresholds at which a pan-coronavirus vaccine is cost saving. As Fig. 3B shows, the pan-coronavirus vaccine only needs an efficacy ≥10% to be cost saving when a 90% efficacious specific vaccine is delayed by 4 months. This threshold decreases to 9% with a 70% efficacious specific vaccine.

## Discussion

Our results show that when the universal vaccine is the only intervention available, it is cost saving even when the efficacy is as low as 10% and coverage is as low as 10% of the U.S. population. Even when a more specific vaccine will become available later in the epidemic, a universal coronavirus vaccine is cost saving as long as the more specific vaccine is delayed by at least 2–5 months, and cost-effective when delayed by at least 2–3 months, depending on the characteristics of the specific vaccine and circulating virus. The universal vaccine also remains cost saving when the cost of vaccinating each person (which includes the price of the vaccine and research and development, storage, distribution, and administration costs of the vaccine) is as high as $110 and cost-effective when the cost of vaccinating each person is as high as $10,390. This suggests that the ceiling for investment into developing and stockpiling such vaccines is rather high. For perspective, the price of COVID-19 vaccines during the pandemic has been $20,^28^ measles, mumps, and rubella (MMR) vaccines cost $24–$89 per dose, pneumococcal vaccines cost $65–$253 per dose, and the shingles vaccine costs $120–$183 per dose.^29^

As can be seen, the efficacy target for a universal coronavirus vaccine does not have to be very high to provide substantial value. This shows the importance of timing and making sure that the population has at least some protection as soon as possible. In other words, if the trade-off is between taking more time to develop a more specific and effective vaccine versus getting a vaccine out as soon as possible, the priority should be speed, and nothing can be faster than a vaccine that has already been developed and stockpiled. This would be especially true for a virus with very high transmissibility (a high R_0_) that could move through the population faster than a more specific vaccine would be available. Although new technological platforms such as mRNA vaccine platforms may allow more rapid development of vaccines, the turnaround time would still be at least 90–120 days for just the manufacturing processing alone.^30^

Our results also alleviate another potential concern about the universal vaccine, that not enough people may be willing to get a more general non-specific vaccine. A coverage threshold of 10% would correspond to 59% of adults 65 years and older, who tend to be at higher risk of more severe clinical outcomes, or 27% of those with hypertension, or 28% of those with obesity. These groups may be different potential target groups when prioritizing vaccination. This is a realistic coverage level to aim for given the fact that primary series and bivalent booster coverage of older adults exceeded 94.5% and 43.3% during the COVID-19 pandemic.^31^ Plus, another advantage of a universal vaccine is that it may be extensively tested and fully approved prior to an epidemic/pandemic, which could enhance vaccine acceptance.

### Limitations

All models, by definition, are simplifications of real-life and cannot account for every possible epidemic scenario and outcome. While we used data from the COVID-19 pandemic to populate, calibrate, and validate the model, sensitivity analyses did show that the pan-coronavirus vaccine remained cost saving through a range of possible R_0_, clinical severity, and vaccine efficacy/coverage values, which can affect the curve of a new epidemic. We aimed to be conservative when determining the value of the pan-coronavirus vaccine by assuming that the strain-specific vaccine efficacy did not wane over time and the strain-specific vaccine did not have more side effects than the pan-coronavirus vaccine (i.e., we assumed the same risk profile of adverse events for each vaccine), stacking the deck against the pan-coronavirus vaccine. Further, we did not account for potential long-term health outcomes such as long COVID-19 and its associated costs, that a pan-coronavirus vaccine may prevent, nor did we consider the use of a pan-coronavirus booster, both of which would increase the clinical and economic value of pan-coronavirus vaccination. The model did specifically represent the U.S. and not other countries. Thus, there is the possibility that different mixing patterns, demographic structures (e.g., different number of people in different age groups), and health outcomes (e.g., differences in care seeking behaviour which can impact costs, treatment, and severity of outcomes) in other countries may affect the value of the pan-coronavirus vaccine. Lastly, the paired t-test assumes normally distributed results, which may be a reasonable assumption given the model includes stochasticity in different parameter value, however, the results may not be normally distributed; we also assume 1000 trials is a random sample of all trials. Further, we performed t-tests with all reported outcomes, and significance may not apply to other outcomes.

Future studies may explore the impact of various coronavirus variants emerging during the course of the epidemic (as was seen with COVID-19), which may offer additional opportunities for the use of a pan-coronavirus vaccine (e.g., if a new strain-specific vaccines needs to be developed for evolving variants). The use of a pan-coronavirus booster could be explored in contexts where such a vaccine's efficacy wanes and there are longer delays in the availability of a strain-specific vaccine. Additionally, future studies may explore the value of a pan-coronavirus vaccine in other countries.

In conclusion, our results show that even at efficacies as low as 10% and coverages as low as 10%, a pan-coronavirus vaccine can be cost-effective and even cost saving as a stand-alone intervention. Even if a highly efficacious strain-specific vaccine becomes available, if it is delayed by at least 3 months, a universal vaccine would be highly cost-effective, becoming cost saving when it is delayed by at least 4 months. This provides support for decision making about stockpiling a pan-coronavirus vaccine for future outbreaks and outlines the vaccine characteristics to aim for in development.

## Contributors

SMB contributed to study design, model conceptualization and development, data collection/literature search, data analysis, accessed and verified the underlying data, interpretation of results, figures, and writing of the manuscript.

KJO contributed to model conceptualization and development, data collection/literature search, data analysis, accessed and verified the underlying data, interpretation of results, figures, and writing of the manuscript.

DCJ contributed to data collection, data analysis, accessed and verified the underlying data, interpretation of results, figures, and writing of the manuscript.

US contributed to study design, data collection/literature search, results interpretation, and manuscript editing.

MEB contributed to study design, data collection/literature search, results interpretation, and manuscript editing.

MFM contributed to model conceptualization, data collection/literature search, interpretation of results, and editing of the manuscript.

AC contributed to study design, interpretation of results, and editing of the manuscript.

KLC contributed to the data analysis, accessed and verified the underlying data, interpretation of results, figures, and editing of the manuscript.

CW contributed to the data analysis, accessed and verified the underlying data, interpretation of results, figures, and editing of the manuscript.

KV contributed to the data analysis, accessed and verified the underlying data, interpretation of results, and editing of the manuscript.

JH contributed to the data analysis, accessed and verified the underlying data, interpretation of results, figures, and editing of the manuscript.

SAS contributed to the interpretation of results and editing of the manuscript.

PJH contributed to study design, data collection, interpretation of results, and editing of the manuscript.

BYL contributed to study conception and design, model conceptualization and development, data analysis, accessed and verified the underlying data, interpretation of results, figures, and writing of the manuscript.

## Data sharing statement

Data used and generated by the model for this study will be available with publication and can be obtained by contacting the authors.

## Declaration of interests

PJH, MEB, and US are co-inventors of a COVID-19 recombinant protein vaccine technology owned by Baylor College of Medicine (BCM) that was recently licensed by BCM non-exclusively and with no patent restrictions to several companies committed to advance vaccines for low- and middle-income countries. The co-inventors have no involvement in license negotiations conducted by BCM. Similar to other research universities, a long-standing BCM policy provides its faculty and staff, who make discoveries that result in a commercial license, a share of any royalty income, according to BCM policy.

SMB, KJO, DCJ, MFM, AC, KLC, CW, KV, JH, SAS, and BYL declare no competing interests.

## Appendix A Supplementary data

Universal SARS Vaccine Appendix_Revision
