# Supplementary material for: The potential epidemiologic, clinical, and economic value of a universal coronavirus vaccine: a modelling study
Source: eClinicalMedicine. 2024 Jan 11;68:102369. doi: 10.1016/j.eclinm.2023.102369 (PMC10965405; doi:10.1016/j.eclinm.2023.102369)
Supplement: Universal SARS Vaccine Appendix_Revision [file mmc1.pdf]

Appendix to: The potential epidemiologic, clinical, and economic value of a universal coronavirus vaccine: a modelling study

Model Validation

Previous publications have described how validation of the model has proceeded.[1-3] For criterion validation, this model has been able to reproduce trends in infection rate, symptomatic cases, hospitalisations, and death rates seen through different phases of the COVID-19 pandemic. For example, when using R_0 2.5 (R_0 corresponding to the original Wuhan strain of SARS-CoV-2) and the probabilities of clinical outcomes observed in the beginning phases of the pandemic, the model generated 100,073,580 infections, 6,739,440 hospitalisations, and 787,375 deaths which aligns with the CDC reported 35,502,419 total infections, 2,420,372 total hospitalisations, and 589,172 deaths, when adjusting for various issues with Centers for Disease Control and Prevention (CDC) data, including data gaps around hospitalisations and deaths (e.g., missing values, unknown data points) and applying the CDC's factors for under-reporting.[4]

Appendix Table 1. Key model input parameters, values, and sources

Parameter	Distribution Type	Mean or Median	Standard Error or Range	Source
**Coronavirus Transmission**				
Latent period (days)	Triangular	5.2	4.1-7.0	5
Infectious period (days)	Uniform	-	3-14	6-9
**Costs (2023 US$)**				
Annual wages (all occupations)	Beta Pert	48,546.78	25,440.38-72,767.13[a]	10
Vaccination (base case)		60	-	11,12
Ambulatory care visit	Uniform	133.66	94.81-188.56	13
Telephone consult	Point Estimate	14.97	-	14
Over the counter medications, daily				
0-12 years old[b]	Gamma	0.11	0.11	15
≥13 years old[c]	Gamma	0.11	0.42	15
Hospitalisation for pneumonia[d]				
0-17 years old	Gamma	28,563.21	36939.59	16
18-44 years old	Gamma	13,227.01	19,580.06	16
45-64 years old	Gamma	16,247.94	20,274.48	16
65-84 years old	Gamma	15,645.24	17,322.53	16
≥85 years old	Gamma	14,717.23	20,614.44	16
Hospitalisation for severe non-pneumonia (all ages)[e]	Gamma	7,525.09	1,292.69	16
Hospitalisation for sepsis[f]				
0-17 years old[g]	Gamma	14,593.29	14,046.36	16
18-44 years old	Gamma	12,289.90	6,582.99	16
45-64 years old	Gamma	23,432.92	23,126.54	16
65-84 years old	Gamma	13,647.72	11,106.37	16
≥85 years old	Gamma	14,593.29	14,046.36	16
Hospitalisation for ARDS[h]				
0-17 years old	Gamma	192,956.48	309,183.65	16
18-44 years old	Gamma	98,658.64	112,090.40	16
45-64 years old	Gamma	90,091.19	134,818.54	16

65-84 years old	Gamma	58,817.21	79,956.93	[16]
≥85 years old	Gamma	48,358.10	45,918.10	[16]
**Probabilities**				
Asymptomatic infection	Triangular	0.35	0.315 - 0.385[i]	[17]
Seroconversion (develop immunity after infection)	Beta Pert	0.760	0.6858-0.8382[i]	[18]
Missing work/school	Point Estimate	1.0	-	Assumption
Ambulatory care	Triangular	0.15	0.06-0.26	[19]
Telephone Consult	-	1-probability of ambulatory care	-	Assumption
Hospitalisation, given infection				
0-17 years old	Beta Pert	0.031	0.028-0.034[i]	[20]
18-44 years old	Beta Pert	0.056	0.051-0.062[i]	[20]
45-64 years old	Beta Pert	0.140	0.126-0.154[i]	[20]
≥65 years old	Beta Pert	0.300	0.270-0.330[i]	[20]
ICU admission, given hospitalisation				
0-17 years old	Beta Pert	0.171	0.154-0.1881[i]	[20]
18-44 years old	Beta Pert	0.152	0.137-0.1675[i]	[20]
45-64 years old	Beta Pert	0.186	0.168-0.2047[i]	[20]
≥65 years old	Beta Pert	0.148	0.133-0.163[i]	[20]
Mortality, given hospitalisation				
0-17 years old	Uniform	-	0.0054-0.0215[j]	[20]
18-44 years old	Uniform	-	0.0186-0.0745[j]	[20]
45-64 years old	Uniform	-	0.0519-0.2076[j]	[20]
≥65 years old	Uniform	-	0.1517-0.6068[j]	[20]
Pneumonia, given hospitalisation	Beta	0.511	0.4599-0.5621	[21]
Sepsis, given hospitalisation				[21,22]
18-44 years old	Beta	0.166	0.1494-0.1826	
≥45 years old	Beta	0.249	0.2241-0.2739	
ARDS, given hospitalisation				[21,23]
18-44 years old	Beta	0.051	0.0459-0.0561	
≥45 years old	Beta	0.169	0.1521-0.1859	
Acute renal failure/acute kidney injury, given hospitalisation				[21,22]
18-44 years old	Beta	0.061	0.0549-0.0671	
≥45 years old	Beta	0.284	0.2556-0.3124	
Acute respiratory failure, given hospitalisation				[21,22]
18-44 years old	Beta	0.374	0.3366-0.4114	
≥45 years old	Beta	0.529	0.4761-0.5819	
**Durations (days)**				
Ambulatory care	Point Estimate	0.5	-	Assumption
Duration of symptoms with mild illness	Triangular	7	3-17	[8,24,25]
Duration of symptoms prior to hospital admission	Triangular	7	3-9[i]	[26,27]
Hospitalisation, not admitted to ICU				
0-49 years old	Gamma	3.9	3.7	[17]
50-64 years old	Gamma	4.9	4.3	[17]
≥65 years old	Gamma	6.2	5.7	[17]
Hospitalisation, ICU (all ages)	Gamma	9	4-17[k]	[28-31]
Hospitalisation, ventilator use	Gamma	9	5-12[k]	[28,30,32]

**Disability weights**			
Mild/moderate illness	0.051	(0.032-0.074)	33
Severe illness	0.133	(0.088-0.190)	33
Critical illness (ICU admission)	0.655	(0.579-0.7127)	34

[a]Values are 95% confidence interval

[b]Assumes 5 to 10 mg/kg of ibuprofen orally every 6 to 8 hours as needed OR 10 to 15 mg/kg of acetaminophen orally every 4 to 6 hours as needed

[c]Assumes 200 mg of ibuprofen or acetaminophen orally every 4 to 6 hours as needed

[d]Uses International Classification of Diseases, Tenth Revision, Clinical Modification (ICD10) code #J13 Pneumonia due to Streptococcus pneumoniae

[e]Uses International Classification of Diseases, Tenth Revision, Clinical Modification (ICD10) code #J11.89 Influenza due to unidentified influenza virus with other manifestations

[f]Uses International Classification of Diseases, Tenth Revision, Clinical Modification (ICD10) code #R65.21 Severe sepsis with septic shock

[g]Data for age-group unavailable and uses lowest values of all age-groups as a proxy

[h]Uses International Classification of Diseases, Tenth Revision, Clinical Modification (ICD10) code #J96.22 Acute and chronic respiratory failure with hypercapnia for 18 years and older and ICD10 code #J96.20 Acute and chronic respiratory failure, unspecified whether with hypoxia or hypercapnia for 0 to 17-year olds

[i]Values are a relative +/- 10% of the mean or median value

[j]Values are interquartile range

[k]Values are 10%-90%

References

1. Bartsch SM, O'Shea KJ, Chin KL, et al. Maintaining face mask use before and after achieving different COVID-19 vaccination coverage levels: a modelling study. *Lancet Public Health* 2022; **7**(4): e356-e65.
2. Bartsch SM, O'Shea KJ, Wedlock PT, et al. The Benefits of Vaccinating With the First Available COVID-19 Coronavirus Vaccine. *American Journal of Preventive Medicine* 2021; **60**(5): 605-13.
3. Bartsch SM, Wedlock PT, O'Shea KJ, et al. Lives and Costs Saved by Expanding and Expediting Coronavirus Disease 2019 Vaccination. *J Infect Dis* 2021; **224**(6): 938-48.
4. Centers for Disease Control and Prevention. COVID-19 Case Surveillance Public Use Data. September 30, 2020. https://data.cdc.gov/Case-Surveillance/COVID-19-Case-Surveillance-Public-Use-Data/vbim-akqf (accessed October 12 2020).
5. Li Q, Guan X, Wu P, et al. Early transmission dynamics in Wuhan, China, of novel coronavirus–infected pneumonia. *New England Journal of Medicine* 2020.
6. Ling Y, Xu S, Lin Y, et al. The persistence and clearance of viral RNA in 2019 novel coronavirus disease survivors. *Chinese Medical Journal* 2020; **133**(9): 1039-43.
7. Wolfel R, Corman VM, Guggemos W, et al. Virological assessment of hospitalized patients with COVID-2019. *Nature* 2020; **581**(7809): 465-9.
8. Chang, Mo G, Yuan X, et al. Time Kinetics of Viral Clearance and Resolution of Symptoms in Novel Coronavirus Infection. *Am J Respir Crit Care Med* 2020; **201**(9): 1150-2.
9. To KK, Tsang OT, Leung WS, et al. Temporal profiles of viral load in posterior oropharyngeal saliva samples and serum antibody responses during infection by SARS-CoV-2: an observational cohort study. *Lancet Infect Dis* 2020.
10. U.S. Bureau of Labor Statistics. Occupational Employment and Wage Statistics. March 31, 2022 2021. https://www.bls.gov/oes/tables.htm (accessed October 27 2022).
11. Centers for Medicare & Medicaid Services (CMS). Medicare COVID-19 Vaccine Shot Payment. 2023. https://www.cms.gov/medicare/covid-19/medicare-covid-19-vaccine-shot-payment (accessed March 10 2023).
12. Centers for Medicare and Medicaid Services. COVID-19 Vaccines and Monoclonal Antibodies. 2023. https://www.cms.gov/medicare/medicare-part-b-drug-average-sales-price/covid-19-vaccines-and-monoclonal-antibodies (accessed March 10 2023).
13. Centers for Medicare & Medicaid Services. Physicians Fee Schedule. January 1, 2022 2022. https://www.cms.gov/medicare/physician-fee-schedule/search/overview (accessed March 10 2022).
14. innoviHealth Systems. Medical coding & billing made easy. 2022. FindACode.com (accessed October 27 2022).
15. IBM. Micromedex RED BOOK. 2022.
16. Agency for Healthcare Research and Quality. National Inpatient Sample (NIS). Healthcare Cost and Utilization Project (HCUP). Rockville, MD: Agency for Healthcare Research and Quality; 2020.
17. Centers for Disease Control and Prevention. COVID-19 Pandemic Planning Scenarios. May 20, 2020 2020. https://www.cdc.gov/coronavirus/2019-ncov/hcp/planning-scenarios.html (accessed June 1 2020).
18. Toh ZQ, Anderson J, Mazarakis N, et al. Comparison of Seroconversion in Children and Adults With Mild COVID-19. *JAMA Netw Open* 2022; **5**(3): e221313.
19. Havers FP, Reed C, Lim T, et al. Seroprevalence of Antibodies to SARS-CoV-2 in 10 Sites in the United States, March 23-May 12, 2020. *JAMA Intern Med* 2020.
20. Stokes EK, Zambrano LD, Anderson KN, et al. Coronavirus Disease 2019 Case Surveillance - United States, January 22-May 30, 2020. *MMWR Morb Mortal Wkly Rep* 2020; **69**(24): 759-65.

21. Owusu D, Kim L, O'Halloran A, et al. Characteristics of Adults Aged 18-49 Years Without Underlying Conditions Hospitalized With Laboratory-Confirmed Coronavirus Disease 2019 in the United States: COVID-NET-March-August 2020. *Clin Infect Dis* 2021; **72**(5): e162-e6.
22. Isath A, Malik AH, Goel A, Gupta R, Shrivastav R, Bandyopadhyay D. Nationwide Analysis of the Outcomes and Mortality of Hospitalized COVID-19 Patients. *Curr Probl Cardiol* 2023; **48**(2): 101440.
23. Suleyman G, Fadel RA, Malette KM, et al. Clinical Characteristics and Morbidity Associated With Coronavirus Disease 2019 in a Series of Patients in Metropolitan Detroit. *JAMA Netw Open* 2020; **3**(6): e2012270.
24. Arashiro T, Furukawa K, Nakamura A. COVID-19 in 2 Persons with Mild Upper Respiratory Symptoms on a Cruise Ship, Japan. *Emerg Infect Dis* 2020; **26**(6).
25. Duszynski T. What Does It Mean to 'Recover From Coronavirus'? Here's What You Need to Know. *Science Alert Health* April 11, 2020. https://www.sciencealert.com/this-is-what-it-means-to-recover-from-corona-and-what-you-can-do-after (accessed.
26. Garg S, Kim L, Whitaker M, et al. Hospitalization Rates and Characteristics of Patients Hospitalized with Laboratory-Confirmed Coronavirus Disease 2019 — COVID-NET, 14 States, March 1–30, 2020. *Morbidity and Mortality Weekly Report* ePub: 8 April 2020.
27. Wang D, Hu B, Hu C, et al. Clinical Characteristics of 138 Hospitalized Patients With 2019 Novel Coronavirus-Infected Pneumonia in Wuhan, China. *JAMA* 2020.
28. Gold JAW, Wong KK, Szablewski CM, et al. Characteristics and Clinical Outcomes of Adult Patients Hospitalized with COVID-19 - Georgia, March 2020. *MMWR Morb Mortal Wkly Rep* 2020; **69**(18): 545-50.
29. Auld SC, Caridi-Scheible M, Blum JM, et al. ICU and Ventilator Mortality Among Critically Ill Adults With Coronavirus Disease 2019. *Crit Care Med* 2020.
30. Bhatraju PK, Ghassemieh BJ, Nichols M, et al. Covid-19 in Critically Ill Patients in the Seattle Region - Case Series. *N Engl J Med* 2020; **382**(21): 2012-22.
31. Lewnard JA, Liu VX, Jackson ML, et al. Incidence, clinical outcomes, and transmission dynamics of severe coronavirus disease 2019 in California and Washington: prospective cohort study. *BMJ* 2020; **369**: m1923.
32. Imam Z, Odish F, Gill I, et al. Older age and comorbidity are independent mortality predictors in a large cohort of 1305 COVID-19 patients in Michigan, United States. *J Intern Med* 2020.
33. Global Burden of Disease Collaborative Network. Global Burden of Disease Study 2019 (GBD 2019) Disability Weights. October 15, 2020 2020 (accessed December 12 2022).
34. Haagsma JA, Maertens de Noordhout C, Polinder S, et al. Assessing disability weights based on the responses of 30,660 people from four European countries. *Popul Health Metr* 2015; **13**: 10.
